# Hospital Economy in War-Time

**Published:** 1915-10-16

**Authors:** 


### HOSPITAL ECONOMY IN WAR-TIME.

We have received from the secretary of an important provincial hospital a return, prepared weekly in his office, from the readings taken by the engineer of the amount of water, gas, and electricity consumed during each week throughout the year. This return enables the management at once to note any abnormal increase in consumption, and whenever this occurs it is promptly investigated, and the check thus established is perfected. A copy of the weekly return, compared with the corresponding week of the preceding year, is displayed in the sisters' sitting-room, which keeps the sisters in touch with the consumption in each department and results in friendly rivalry to maintain the most economical returns. We annex the comparative return for the week ending September 27, 1914, and for the weeks ending September 20 and 27, 1915.

*Comparative Statement of Water, Gas, and Electricity used during the weeks ending September* 20 *and* 27, 1915, *and September* 27, 1914.

| | 1915. Sept. 20 | 1915. Sept. 27 | 1914. Sept. 27 |
|---|---|---|---|
| **Water, in Gallons.** | | | |
| No. 1 Meter (Porter's House, Mortuary, and Laundry when using hard water) ... ... | 15,300 | 16,100 | 11,200 |
| No. 2 Meter (Wards Water Softener and Laundry) ... ... | 60,600 | 61,500 | 52,100 |
| | 75,900 | *77,600* | 63,300 |
| No. 3 Meter (Administration Block) ... | 5,600 | 2,500 | 5,900 |
| No. 4 Meter (Nurses' Home) ... ... ... | 6,000 | *9,100* | 5,170 |
| No. 5 Meter (Out-Patients) ... ... | 1,000 | 800 | 2,400 |
| **Gas, in Cubic Feet.** | | | |
| Kitchen ... ... ... | 9,900 | 9,200 | 10,800 |
| No. 1 Ward ... ... | 2,700 | 2,400 | 2,100 |
| No. 2 Ward ... ... | 2,400 | 2,400 | 1,500 |
| Out-patients ... ... | 800 | *900* | 500 |
| Laundry ... ... ... | 3,000 | *3,300* | 4,000 |
| No. 3 Ward ... ... | 4,000 | *4,200* | — |
| **Electric Light, in Units.** | | | |
| Kitchen ... ... ... | 3 | 3 | 3 |
| No. 1 Ward ... ... | 10 | *14* | 11 |
| No. 2 Ward ... ... | 14 | 14 | 11 |
| No. 4 Ward ... ... | 13 | 13 | 7 |
| No. 5 Ward ... ... | 5 | *22* | 9 |
| No. 6 Ward ... ... | 10 | *11* | — |
| No. 7 Ward ... ... | 15 | 13 | 17 |
| Out-patients ... ... | 2 | *3* | 3 |
| Laundry and No. 3 Ward ... ... ... | 31 | *36* | 15 |
| Nurses' Home ... ... | 10 | *11* | 10 |
| **Electric Power, in Units.** | | | |
| Pumps Lift and X-ray ... | 175 | 164 | 216 |

In considering the figures it may be well to bear in mind that the hospital has had an increased number of patients in 1915, owing to the accommodation provided for sick and wounded soldiers. We should welcome any comment on the plan pursued and any suggestions for its improvement.

